# Development and validation of a physical frailty phenotype index-based model to estimate the frailty index

**DOI:** 10.1186/s41512-023-00143-3

**Published:** 2023-03-21

**Authors:** Yong-Hao Pua, Laura Tay, Ross Allan Clark, Julian Thumboo, Ee-Ling Tay, Shi-Min Mah, Pei-Yueng Lee, Yee-Sien Ng

**Affiliations:** 1 Department of Physiotherapy, Singapore General Hospital, Outram Road, Singapore, 169608 Singapore; 2 Medicine Academic Programme, Duke-NUS Graduate Medical School, Singapore, Singapore; 3 Department of General Medicine (Geriatric Medicine), Sengkang General Hospital, Singapore, Singapore; 4 School of Health and Behavioural Science, University of the Sunshine Coast, Sunshine Coast, Australia; 5 Department of Rheumatology and Immunology, Singapore General Hospital, Singapore, Singapore; 6 Health Services Research & Evaluation, SingHealth Office of Regional Health, Singapore, Singapore; 7 Department of Physiotherapy, Sengkang General Hospital, Singapore, Singapore; 8 Organization Planning and Performance, Singapore General Hospital, Singapore, Singapore; 9 Geriatric Education and Research Institute, Singapore, Singapore; 10 Duke-NUS Medical School, Singapore, Singapore; 11 Department of Rehabilitation Medicine, Singapore General Hospital and Sengkang General Hospital, Singapore, Singapore

**Keywords:** Frailty scales, Frailty phenotype, Frailty index, Geriatrics, Prediction

## Abstract

**Background:**

The conventional count-based physical frailty phenotype (PFP) dichotomizes its criterion predictors—an approach that creates information loss and depends on the availability of population-derived cut-points. This study proposes an alternative approach to computing the PFP by developing and validating a model that uses PFP components to predict the frailty index (FI) in community-dwelling older adults, without the need for predictor dichotomization.

**Methods:**

A sample of 998 community-dwelling older adults (mean [SD], 68 [7] years) participated in this prospective cohort study. Participants completed a multi-domain geriatric screen and a physical fitness assessment from which the count-based PFP and the 36-item FI were computed. One-year prospective falls and hospitalization rates were also measured. Bayesian beta regression analysis, allowing for nonlinear effects of the non-dichotomized PFP criterion predictors, was used to develop a model for FI (“model-based PFP”). Approximate leave-one-out (LOO) cross-validation was used to examine model overfitting.

**Results:**

The model-based PFP showed good calibration with the FI, and it had better out-of-sample predictive performance than the count-based PFP (LOO-*R*^2^, 0.35 vs 0.22). In clinical terms, the improvement in prediction (i) translated to improved classification agreement with the FI (Cohen’s *k*_w_, 0.47 vs 0.36) and (ii) resulted primarily in a 23% (95%CI, 18–28%) net increase in FI-defined “prefrail/frail” participants correctly classified. The model-based PFP showed stronger prognostic performance for predicting falls and hospitalization than did the count-based PFP.

**Conclusion:**

The developed model-based PFP predicted FI and clinical outcomes more strongly than did the count-based PFP in community-dwelling older adults. By not requiring predictor cut-points, the model-based PFP potentially facilitates usage and feasibility. Future validation studies should aim to obtain clear evidence on the benefits of this approach.

**Supplementary Information:**

The online version contains supplementary material available at 10.1186/s41512-023-00143-3.

## Background

With the fast-aging population worldwide, accurate screening for individuals early in their trajectory towards frailty is an urgent and unmet need [1]. Over 60 frailty instruments have been developed to measure frailty amongst which the Cardiovascular Health Study (CHS) physical frailty phenotype (PFP) [2] and the frailty index (FI) [3] are widely used [4]. The multi-dimensional FI measures frailty by the accumulation of deficits across the domains of medical health and physical, social, and cognitive functioning. As a continuous measure, the FI is a sensitive measure of frailty [5, 6]. However, comprising at least 30 deficit items, the FI may not be suitable for large-scale frailty screening. The PFP measures frailty by assessing 5 biologic manifestations of frailty that are primarily physical in nature—that is, reduced gait speed, muscle strength, body mass, physical activity, and energy levels. The PFP is constructed by dichotomizing these 5 criterion predictors and summed to produce a count-based measure. Presumably, this dichotomization approach to creating a PFP count score facilitates ease-of-use and clinical interpretability; however, it has limitations.

First, a count-based approach assumes that the PFP criterion predictors weigh equally—an assumption that may be invalid in light of findings that individual predictors may have varying prognostic or predictive associations with FI [5, 7] and clinical outcomes [8, 9]. Second, constructing the PFP score, originally described by Fried et al. [2], necessitates dichotomizing its criterion predictors using the 20th percentile population cut-point. However, the appropriate reference population data are often not available in many settings, thereby reducing the feasibility of the PFP [10]. In the absence of population-specific cut-points, a population-independent or literature-derived cut-point approach has been advocated and widely adopted [11]. However, for a given PFP criterion (e.g., gait speed), several cut-points have been proposed in the literature [11–15], potentially resulting in varying prevalence estimates of prefrailty/frailty which hinder harmonization and comparison of findings.

Third, dichotomization discards information and decreases the discrimination power of the predictors [16]. This information loss leads to assumptions that are clinically unrealistic. For example, predictor dichotomization assumes participants with similar gait speed values on opposite sides of a 1.0m/s cut-point—for example, 0.95m/s and 1.05m/s—are classified differently as having “slow” and “normal” gait speed, respectively. Given these assumptions, the ability of the count-based PFP to finely grade the degree of frailty is likely to be adversely affected.

Taken altogether, a count-based dichotomization approach reduces the full predictive potential of the PFP, which may partially explain why (i) the FI had reportedly at least comparable but often better predictive performance than the PFP [7, 17–19] and (ii) the PFP was reportedly less adept than the FI in discriminating levels of frailty particularly at the early stages of frailty [6, 19]. Furthermore, we believe it is possible for the often-reported poor-to-fair classification agreement [7, 18–20] between the PFP and FI to be attributed not only to the conceptual differences between the 2 instruments but also to the discrimination loss from predictor dichotomization.

Against this background, we propose a more feasible approach to computing the PFP by developing and validating a regression model for FI in community-dwelling older adults using criterion predictors of the PFP (termed “model-based PFP” henceforth). Specifically, (i) analyzing the FI as the response variable capitalizes on its continuous nature [5] whilst (ii) analyzing the PFP components as continuous (or ordinal) variables in the regression model overcomes problems of information loss and arbitrary predictor stratification using cut-points that have tended to vary across time and studies.

## Methods

### Participants and procedures

This prospective cohort study comprised 998 community-dwelling ambulant adults aged ≥50 years who participated in “Individual Physical Proficiency Test for Seniors” (IPPT-S)—an ongoing community-based program designed to promote fitness and to prevent or delay sarcopenia and frailty in older adults [21]. The institutional review board approved the study (SingHealth CIRB 2018/2115, Singapore), and all participants provided written informed consent. Consenting participants completed a questionnaire-based multi-domain geriatric screen and a physical fitness assessment at baseline assessment, and they were followed up 1 year later via telephone interview.

### Frailty index (FI)

The 36-item FI was constructed following a standardized procedure which included medical comorbidities, functional performance deficits, cognitive and sensory impairments, and psychosocial problems [3] (Additional file 1: Appendix A details the FI items and their associated scores.) The FI is the proportion of deficits present and similar to previous studies [7, 19], and the participants were classified as being robust (≤0.10), pre-frail (>0.10–0.21), and frail (>0.21).

### Physical frailty phenotype

The modified PFP comprised the 5 criteria of (i) slowness, (ii) weakness, (iii) shrinking, (iv) low physical activity, and (v) exhaustion. The “slowness” criterion was measured by the 10-m habitual gait-speed test, and slow gait speed was defined by a cut-point of <1.0 m/s [13, 22]. The “weakness” criterion was measured by the handgrip strength test, which was measured using a Jamar digital dynamometer (Sammons USA), and the testing procedures followed the Southampton protocol [23]. Consistent with recent recommendations [4], the maximal reading from all trials (2 trials for each hand) was analyzed, and weak handgrip strength was defined using cut-points of <28 kg for men and <18 kg for women [22]. The “shrinking” criterion was defined by a body mass index (BMI) of ≤18.5kg/m^2^ [24].

The “low physical activity level” criterion was measured by the total walking time per week (hours/week). Notably, physical activity was operationally defined by walking time—the most common form of physical activity amongst older adults [25]—to facilitate external validation of the model-based PFP in established studies that have tended to use different physical activity questionnaires. In our study, low physical activity level was defined by a total waking time < 2 h (or 120 min)/week [26]. Finally, the “exhaustion” criterion was measured by 2 questions about effort and motivation from the Center for Epidemiological Studies-Depression Scale [27].

The count-based PFP was graded using the number of criteria satisfied, and the participants were classified as being robust (0 criterion), pre-frail (1-2 criteria), and frail (≥3 criteria) [2]. For the model-based PFP, the PFP component criteria and sex were included in a Bayesian model which generated a continuous FI measure (described later), from which the 3 frailty categories could be derived using FI-defined cut-points (Table 1 details the operational definitions).

**Table 1:** Operational definition of count- and model-based physical frailty phenotype

Criteria	Count-based PFP (modified)	Model-based PFP
Slowness	Habitual gait speed was measured based on time taken to walk 10m at comfortable pace. Based on existing guidelines, slowness was defined by a gait speed of <1.0 m/s.	Gait speed was modeled continuously and nonlinearly using thin plate regression splines.
Weakness	Maximum handgrip strength measured using a handheld dynamometer. Weakness was defined using the AWGS 2019 cutoffs: handgrip strength <28 kg for men and <18 kg for women	Handgrip strength was modeled continuously and nonlinearly using thin plate regression splines. Instead of using gender-specific cut-points, gender was included in the model
Shrinking	Consistent with previous work, shrinking was defined by a body mass index (BMI) of <18.5kg/m^2^	Both body weight and height were modeled continuously and nonlinearly using thin plate regression splines
Low physical activity level	To facilitate comparability between studies that used different physical activity scales, physical activity level was measured by the total walking time per week based on the product of the self-reported frequency of walking per week (0-7days) and duration of walking per day (mins/day). Low physical activity level was defined by a total waking time < 2 hours or 120mins/week	Total walking time (hours/week) was cubic root transformed and modeled nonlinearly using thin plate regression splines.
Exhaustion	Exhaustion was measured by 2 items of the Centre for Epidemiological Studies-Depression Scale (CES-D): (Q1) I felt that everything I did was an effort and (Q2) I could not get going. Exhaustion was defined by answering at least “a moderate amount of the time” to either question	Both CES-D items were modeled as monotonic predictors.
Scoring	Each criterion yields a dichotomous score of 0 or 1. Count-based PFP was the sum of criteria and it classified patients as robust (0), prefrail (1–2), and frail (3–5).	PFP component criteria and gender were included in a Bayesian model which generated a continuous FI measure, from which the 3 frailty categories could be derived: robust (≤0.10), pre-frail (>0.10–0.21), and frail (FI>0.21)

*PFP* physical frailty phenotype, *CES-D* Centre for Epidemiological Studies-Depression Scale, *FI* frailty index, *AWGS* Asian Working Group for Sarcopenia

### Clinical outcomes

Clinical outcomes were self-reported (i) incident falls resulting in emergency department visits and (ii) all-cause hospitalization within 1 year after baseline assessment.

### Statistical analysis

We used means with SDs and medians with IQRs for continuous variables and frequencies with percentages for categorical variables. Amongst those with non-missing FI, all PFP criterion predictors were missing at very low levels (0.2 to 1.5%). Thus, we used the *transcan* function in the *Hmisc* [28] R package to singly impute missing values.

### Model and prior specification

To develop the model-based PFP, we fitted a Bayesian multivariable beta regression model, which included (i) FI as the response variable and (ii) PFP component criteria and sex as predictors. A Bayesian analytical framework was used because it aligned closely with our objectives of (i) modeling the 2 PFP “exhaustion” criterion items flexibly as monotonic ordered predictors [29] and (i) providing interpretable uncertainty estimates of the predicted FI values. Beta regression was used because it is a flexible approach to model the FI—a continuous proportion with a non-normal distribution [30]. The model-based PFP was reported according to the transparent reporting of a multivariable prediction model for Individual Prognosis or Diagnosis (TRIPOD) guidelines [31].

Our goal was to optimize the predictive accuracy of the PFP by preserving information in its criterion predictors. Thus, gait speed, handgrip strength, body weight, body height, and total walking time were treated as continuous variables. For total walking time, this variable was first transformed using its cube root to reduce the potential influence of extreme values. To allow prior distributions to be scaled for other predictors, we standardized them as *z* scores. To avoid assuming linearity for all continuous predictors, we modeled them with thin-plate splines [32]. For the 2 “exhaustion” variables, we modeled these ordinal predictors using the “monotonic effects” function [29] which allows ordinal categories to exert individual conditional effects whilst maintaining monotonically (same directionality).

In our analyses, we set weakly informative prior distributions for the model parameters to reduce the likelihood of estimating unrealistic values without excluding reasonable values [33]. All Bayesian models were fitted using *Stan* [34] through the *brms* [35] R package. Stan implements the Hamiltonian Monte Carlo with No-U-Turn sampling algorithm [34], and each model used 4 chains, 3000 iterations per chain, to generate the posterior samples for all parameters (Additional file 1: Appendix B provides the model implementation details.). From these samples, we derived the posterior predictive distribution of the FI which could be interpreted as the predictions of possible mean FI values for a given individual characterized by a given set of PFP criterion values. To summarize this distribution, we used mean as point estimate and 95% credible interval (CrI) as the interval with 95% probability of containing the true FI, given our prior knowledge and observed data.

### Model performance

To evaluate the model-based PFP in relation to current practice, we developed a “referent” model that had the PFP count score as its only predictor. Given that gait speed was, amongst the PFP criterion predictors, reportedly the strongest predictor of FI and clinical outcomes [5, 7–9], we also fitted a “gait speed” model which included gait speed and covariates [5] routinely and easily obtained in the clinical setting–namely, age, sex, body weight, and body height. To evaluate whether the performance of the model-based PFP could simply be the result of overfitting a more complex model, we used approximate Bayesian leave-one-out (LOO) cross-validation—a technique that assesses how well a model potentially generalizes to new individuals [36]. Notably, the approximate LOO cross-validation technique, based on Pareto smoothed importance sampling [36], was chosen because the full LOO cross-validation process is computationally burdensome in the Bayesian setting. Accordingly, for all models, we computed (i) their respective approximate LOO cross-validated *R*^2^ (LOO-*R*^2^) and (ii) the paired difference between their respective approximate LOO cross-validated expected log-predictive density (denoted using ELPD_diff_). Notably, as ELPD_diff_ was estimated with respect to the best-performing model, an absolute ELPD_diff_ of greater than twice its standard error was taken as evidence that the best-performing model had better out-of-sample predictive performance than the alternative model. Finally, we evaluated calibration of the referent model and the model-based PFP using locally weighted scatterplot smoothing calibration plots.

### Classification performance

To assess agreement of the various models with the ordinal FI-defined frailty categories, we stratified participants by their mean posterior predicted FI values into robust (posterior predicted FI≤0.10), pre-frail (>0.10–0.21), and frail (FI>0.21), and we computed Cohen’s quadratic weighted kappa coefficient. To assess discriminative performance, we compared the ability of count- and model-based PFP to identify participants with FI-defined prefrailty/frailty (FI>0.10) using the area under the receiver-operating characteristics curve (AUC) with DeLong’s test. To assess the clinical relevance of the improvement in discriminative performance over the count-based PFP, we computed the categorical net reclassification index (NRI) statistic [37]. To provide a clinical view on the consequences of reclassification, similar to previous analyses [20], we compared participants with discrepant frailty classifications by FI and PFP on their demographic and clinical characteristic variables.

### Prognostic performance

To assess prognostic performance of the count- and model-based PFP in predicting clinical outcomes, we fitted separate binary logistic regression models for 1-year incident falls and hospitalization. In these models, count-based PFP was modeled as a count variable whilst model-based PFP was modeled as a continuous variable based on the posterior predicted FI values. The AUCs of the models were compared using the DeLong’s test. To evaluate whether model-based PFP provided incremental prognostic information over the conventional count-based PFP, we compared nested binary logistic regression models with a likelihood ratio *χ*^2^ test. To summarize its added prognostic value, we computed the proportion of explainable variation that was explained by the model-based PFP (calculated as 1 minus the ratio of variances of predicted values before and after adding model-based PFP to the model containing only count-based PFP) [38]. In all analyses, we have chosen to perform complete-case analyses because (i) we did not have strong auxiliary outcome variables for multiple imputation and (ii) we have observed that the baseline characteristics of participants without outcome data were similar to those of participants with outcome data (Additional file 1: Appendix C).

## Results

### Demographics

Table 2 shows that the mean age of all 998 participants was 68 years (SD, 6) and women accounted for accounted for nearly three-quarters (74%) of the sample. Based on the FI, 49% (*n*=485) of participants had pre-frailty/frailty based on the count- and model-based PFP, 38% and 55%, respectively. At 1-year follow-up, 561 patients (56%) completed at least a telephone interview and incidence rates for falls and all-cause hospitalization were 14% and 12%, respectively.

**Table 2:** Sample characteristics

Variables	Values
Age (years)	63 **67** 72 (67.6 ± 7)
Women	74% (741)
Weight (kg)	52 **59** 67 (60 ±12)
Height (m)	1.51 **1.56** 1.61 (1.56 ±0.08)
BMI (kg/m^2^)	21.7 **23.9** 26.8 (24.5 ± 4.5)
Living alone	18% (179)
Impaired cognitive performance^a^	7% (71)
Hearing difficulty	16% (162)
Vision difficulty	20% (195)
Hypertension	45% (446)
Diabetes mellitus	19% (194)
Depression^b^	15% (146)
Arthritis	18% (179)
Ischaemic heart disease	3% ( 30)
Stair climbing difficulty	16% (160)
Lifting (10pounds) difficulty	17% (169)
Frailty index (FI)	0.06 **0.10** 0.15 (0.11 ±0.07)
FI classification	
Robust (≤0.10)	51% (513)
Prefrail (>0.10-0.21)	38% (383)
Frail (>0.21)	10% (102)
Count-based PFP	
Robust (0pts)	62% (620)
Prefrail (1-2pts)	35% (347)
Frail (3-5pts)	3% ( 31)
Model-based PFP	
Predicted FI	0.08 **0.10** 0.13 (0.11 ±0.04)
Robust (≤0.10)	45% (452)
Prefrail (>0.10-0.21)	51% (506)
Frail (>0.21)	4% ( 40)

Continuous variables are summarized as the 25th, 50th, 75th percentiles (mean ± SD). Categorical variables are summarized as percentages and frequencies (*N*)

*FI* frailty index, *PFP* physical frailty phenotype

^a^Modified Chinese version of Mini Mental State Examination (cMMSE) < 21 points

^b^Geriatric Depression Scale (GDS) ≥5 points

### Predictive performance

**Fig. 1:**
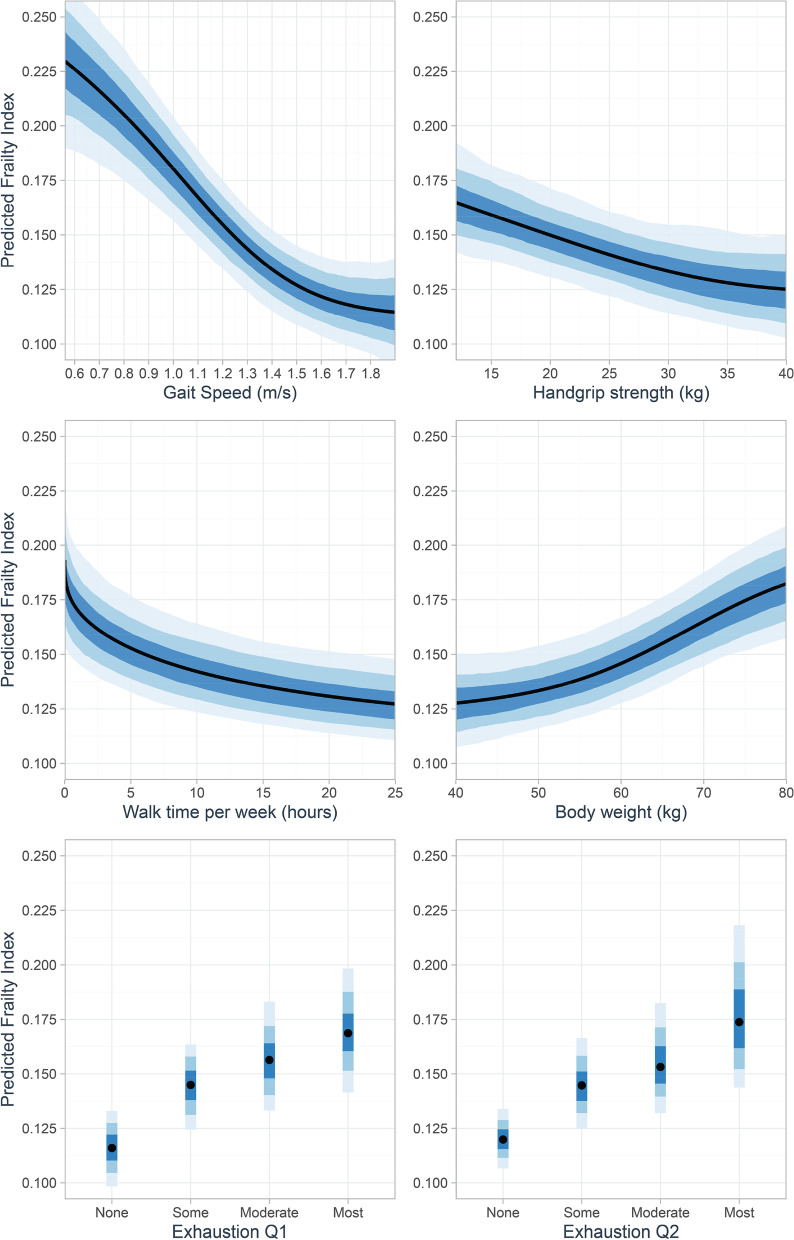
Multivariable associations (black lines or points) of physical frailty phenotype criterion predictors (expressed on their natural scales for interpretability) with Frailty Index. Predicted mean frailty index values were calculated from a Bayesian beta regression model using thin-plate splines for continuous predictors and the monotonic effects approach for ordinal predictors. For all predictors, ribbons are 95% (light blue), 80% (medium blue), and 50% (dark blue) credible intervals

**Fig. 2:**
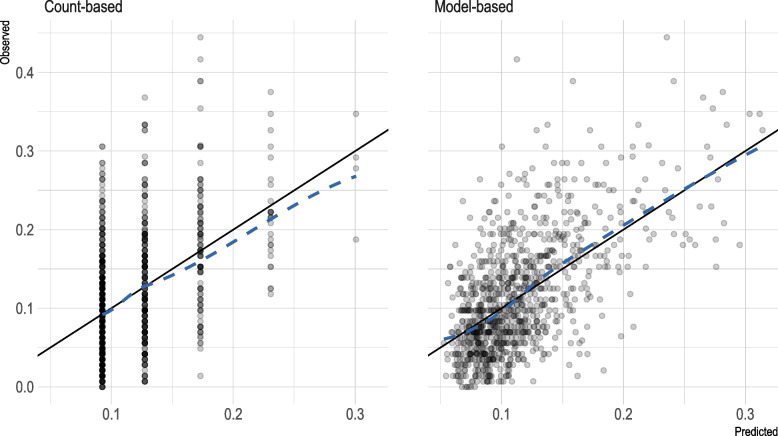
Visual assessment of model calibration for frailty index (FI). Predicted FI were derived from a model using the count-based physical frailty phenotype (PFP) as the only predictor (left panel) and a model using non-dichotomized PFP criterion predictors (right panel). Solid line represents the identity line. Dotted line represents a lowess smoother through the data points, showing good calibration (linear relation) between observed and predicted FI values for the model-based PFP

All models converged and the LOO cross-validation process was reliable with all Pareto *k* values below 0.5. (Additional file 1: Appendix D shows the trace plots of all model parameters). For the model-based PFP, all PFP criterion predictors were predictive of FI and Fig. 1 shows their multivariable associations—including nonlinear relations—with FI. Overall, the model-based PFP had better predictive performance (LOO-*R*^2^, 0.35; Table 3) than either the referent model containing the count-based PFP (0.22) or the gait speed model (0.26). Formal model validation and comparison using approximate LOO cross-validation showed that the model-based PFP potentially generalized to new individuals better than the referent model (ELPD_diff_ [SE] = −91 [15]) and the gait speed model (ELPD_diff_ [SE] = −51 [13]). Besides having better predictive performance, the model-based PFP showed good calibration with the observed FI (Fig. 2).

**Table 3:** Model performance and classification accuracy statistics^a^

	Referent model	Gait speed model	Model-based PFP
**Model performance (95% CrI)**			
*R*^2^	0.22 (0.17 to 0.27)	0.27 (0.22 to 0.32)	0.37 (0.32 to 0.41)
**Approximate LOO cross-validation**			
LOO-*R*^2^	0.22 (0.17 to 0.27)	0.26 (0.21 to 0.31)	0.35 (0.29 to 0.40)
LOO-ELPD_diff_ (SE)^b^	−90.9 (14.9)	−50.7 (12.6)	0.0
**Classification performance (95% CI)**			
Cohen kappa^c^	0.36 (0.30 to 0.42)	0.40 (0.35 to 0.45)	0.47 (0.42 to 0.52)
AUROC^d^	0.67 (0.64 to 0.69)	0.74 (0.71 to 0.77)	0.77 (0.74 to 0.80)
Overall NRI^d^	-	0.05 (−0.02 to 0.13)	0.11 (0.05 to 0.18)
Event NRI	-	0.20 (0.14 to 0.25)	0.23 (0.18 to 0.27)
Non-event NRI	-	−0.14 (−0.09 to −0.19)	−0.11 (−0.07 to −0.16)

*Abbreviations: PFP* Physical Frailty Phenotype, *CrI* credibility interval, *CI* confidence interval, *SE* standard error, *LOO-CV* leave-one-subject-out cross-validation, *LOO-R*^*2*^ leave-one-subject-out R-squared statistic, *ELPD*_*diff*_ pairwise difference in leave-one-out expected log posterior density, *AUROC* area under the receiver operating characteristic curve, *NRI* net reclassification index

^a^Model performance of the model-based physical frailty phenotype (PFP) (a model with non-dichotomized PFP criterion predictors) was compared to that of the referent model (a model with only the PFP count score) and the gait speed model (a model with gait speed and standard covariates)

^b^Pairwise difference in leave-one-out (LOO) expected log posterior density (denoted using ELPD_diff_) between models and its standard error (SE). As ELPD_diff_ was estimated with respect to the best-performing model, an absolute ELPD_diff_ of greater than twice its SE was taken as evidence that the best-performing model (with a ELP_diff_ of 0) had better out-of-sample predictive performance than the alternative model

^c^Cohen’s quadratic-weighted kappa coefficients computed based on frailty index-defined robust (≤0.10), pre-frail (>0.10 to 0.21), and frail (>0.21) categories

^d^AUROC (area under the receiver operating characteristic curve) and net reclassification index (NRI) computed based on frailty index-defined robust (≤0.10) and pre-frail/frail (>0.10) categories

### Classification performance

In terms of classification agreement, frailty classifications by the count-based PFP showed fair agreement with those by FI (*kw*= 0.36; 95%CI, 0.30 to 0.42) whilst model-based PFP showed greater (moderate) agreement (*kw*= 0.47; 95%CI, 0.42 to 0.52). In terms of model discrimination ability to separate participants with and without FI-defined prefrailty/frailty, the AUROC for the model-based PFP (0.77, 95% CI, 0.74 to 0.80) was higher than the count-based PFP (0.67; 95% CI, 0.64to 0.69; Delong’s *P*<0.001). In terms of the ability of the model-based PFP to correctly reclassify participants over the count-based PFP, as shown in Table 3 and Additional file 1: Appendix E, model-based PFP resulted in a 23% (110/484) net increase in FI-defined “prefrail/frail” participants correctly classified (event NRI, 0.23; 95% CI, 0.18 to 0.27) but a 11% (59/514) net loss in FI-defined “robust” participants correctly classified (non-event NRI, −0.11; 95% CI, −0.07 to −0.16), with an overall NRI of 0.11 (95% CI, 0.05 to 0.18). Across all performance metrics, the referent model did not outperform the gait speed model, with the latter model having potentially better model generalizability (ELPDdiff [SE] comparing gait speed vs referent models = −40 [10]) and discrimination ability (AUROC, 0.72; *P*<0.05).

### Prognostic performance

Overall, the model-based PFP showed stronger prognostic performance than count-based PFP in predicting incident falls and hospitalization. When predicting the risk of incident falls, the model-based PFP had an AUC of 0.56 whist the count-based PFP had an AUC of 0.51 (*P*=0.10 for difference between 2 models). Using the likelihood ratio *χ*^2^ test for nested models, the model-based PFP predictor added statistically significant incremental predictive value (*P*<0.01) to a model comprising the conventional count-based PFP predictor. In a model comprising both predictors, ~93% of its prognostic information was attributed to the model-based PFP predictor. When predicting the risk of incident hospitalization, the model-based PFP showed higher AUC (0.63 vs 0.55; *P=*0.01) and it provided statistically significant incremental prognostic value above count-based PFP (*P*<0.01). In a model comprising both predictors, ~87% of its prognostic information was attributed to the model-based PFP predictor**.**

## Discussion

In 998 community-dwelling older adults, we developed a model-based PFP which showed better prognostic performance for clinical outcomes and predicted FI more accurately than the count-based PFP. Specifically, a modeling approach that (i) avoided dichotomizing the PFP criterion predictors and (ii) avoided assuming that predictors act equally or linearly better captured the relationship between the PFP and FI (LOO-*R*^2^, 0.35 vs 0.22). In clinical terms, the improvement in prediction translates to improved classification agreement with the FI (*k*_w_, 0.47 vs 0.36) and an overall net correct reclassification of 11% for FI-defined prefrailty/frailty. Importantly, model validation using approximate LOO cross-validation indicated that this improvement in predictive and classification performance was unlikely to be achieved by over-fitting a more complex model. Overall, our findings of lower predictive and classification accuracy for the count-based PFP are consistent with those from both clinical [39] and simulation [40] studies demonstrating the substantial loss of information and predictive power from predictor dichotomization. Indeed, our count-based PFP comprising 5 elaborately-obtained—but eventually dichotomized—criterion predictors did not even outperform a model comprising a non-dichotomized gait speed predictor and standard covariates, further attesting to the toll of dichotomization.

Dichotomizing the criterion predictors to create the count-based PFP requires the availability of a contemporary reference population, from which the lowest quintile cut-points derive [2]. In the absence of population normative data, several cut-points have been proposed in the literature even for the same criterion. For example, proposed cut-points for gait speed have included 0.8m/s [11, 12], 0.9m/s [14], 1.0m/s [13], and 1.1m/s [15]. Collectively, these cut-points led to the question: Do optimal cut-points exist? In our analyses, we allowed potential nonlinear effects for the criterion predictors, and we found that whilst nonlinear in form (Fig. 1), their associations with FI did not evince sharp inflection points which argue against the existence of universal cut-points. In the absence of apparent thresholds, recent simulation [40] and clinical [41] studies have indicated that it is unlikely for the study-specific predictor cut-points to generalize. Thus, although our findings await further confirmation, we believe the concept of population-independent cut-points should be interpreted with some caution. Consistent with previous recommendations [40, 41], we urge future studies aspiring to propose new optimal predictor cut-points to first inspect the relationship between the PFP criterion predictors and various clinical outcomes and explore whether optimal thresholds are apparent.

In our study, classification agreement between count-based PFP and FI was fair (*k*_*w*_ = 0.36)—a finding consistent with several previous studies [7, 18–20]. When compared to previous studies [7, 18, 20], another consistent finding was that amongst participants with discrepant frailty classifications, proportionally more were classified as prefrail/frail by the FI (228 vs 112; Additional file 1: Appendix F1). Different from previous studies, however, our findings shed further light by showing that classification agreement improved to moderate (*k*_*w*_ = 0.47) with the model-based PFP. Amongst participants with discrepant frailty classifications, participants classified as prefrail/frail by the FI but not by the model-based PFP substantially reduced in number (*n*=181 vs 228) and they were less likely to report having stair climbing difficulties (Additional file 1: Appendix F2). Given this improvement in sensitivity (event NRI, 23%; Table 3), the model-based PFP may be less prone to the criticism often made of the PFP—that the (count-based) PFP may be less adept than the FI in discriminating levels of frailty particularly at the early stages of frailty [6, 19]. Further studies are needed to confirm the improved sensitivity of model-based PFP over the count-based PFP.

Besides predictive and discriminative accuracy, ease-of-use and result interpretability are keys to adoption and implementation. Although model complexity and ease-of-use are often seen as competing factors, we argue that they need not be trade-offs. Indeed, whilst the flexible modeling of predictors and the inclusion of spline terms may have complicated the underlying algorithm of the model-based PFP, this approach has removed the need for predictor cut-points which likely facilitates usage and feasibility. Furthermore, to promote ease-of-use, we have incorporated the model into an online calculator (https://sghpt.shinyapps.io/ippts_pfp/), and the approximated model equation can be found in Additional file 1: Appendix G. To facilitate results interpretability, we have used (i) a Bayesian modeling framework to generate continuous predicted FI scores and (ii) established FI cut-points to generate frailty classifications based on the predicted FI scores. Given this flexibility and depending on the context and purpose, the model-based PFP could potentially be used as a continuous variable for prediction and longitudinal tracking purposes or as a categorical variable for risk-stratification purposes. That said, we should clearly state that the model-based PFP was developed into an online calculator purely as a proof-of-concept and a thought-starter for encouraging similar validation work across the diverse populations and settings where both PFP and FI measures have already been collected. Hence, pending external validation, its use should be confined to research purposes at present.

### Limitations

Our study has limitations. First, the model-based PFP was developed and validated in Asian older adults; hence, it may not directly apply to non-Asians. Nonetheless, our study should be rightly viewed as a proof-of-concept for the potential use of the model-based PFP, and we hope it will encourage similar work in other racial/ethnic groups. Second, our use of the FI as the reference standard may be criticized as it is not the gold standard frailty measure. In the absence of a gold standard, however, we believe the FI is a sensible choice because of (i) its continuous nature, (ii) its positive association with the count-based PFP [7, 42], and (iii) and its comparable—and if not often better—predictive performance than the count-based PFP [7, 17–19]. Third, we did not have follow-up clinical outcomes of 44% of the participants; however, included and excluded participants did not differ meaningfully in baseline characteristics and frailty status (Additional file 1: Appendix C). Whilst our analyses focused on the relative prognostic performance of the count-based and model-based PFP, it is unknown how the missing data would impact the results. Fourth, we validated the model-based PFP using approximate LOO cross-validation but this strategy could be criticized for not representing a true external validation procedure performed in samples geographically and temporally different from our development sample. Nonetheless, given the current study findings and existing knowledge about the limitations of predictor dichotomization, we expect the model-based PFP to have better predictive and classification performance than the count-based PFP in other settings.

## Conclusions

In community-dwelling older adults, we developed and validated a model-based PFP which predicted adverse clinical outcomes and FI more strongly than did the count-based PFP. By not needing population-specific predictor cut-points, the model-based approach represents a potentially feasible and innovative method to compute the PFP. As many cohort studies have obtained both PFP and FI measures, it is our hope that this work could efficiently leverage on existing studies to further evaluate the model-based PFP. Future work should also aim to obtain clear evidence on the benefits of this model-based approach compared with the conventional count-based approach.

## Supplementary Information

**Additional file 1: Appendix A.** 36-Item frailty index. **Appendix B.** Model implementation details for model-based PFP. **Appendix C.** Demographic and clinical characteristics of participants with and without one-year follow-up clinical outcomes. **Appendix D.** Markov Chain Monte Carlo (MCMC) simulation diagnostics. **Appendix E.** Reclassification of participants by net reclassification index (NRI) with use of gait speed model or model-based PFP versus count-based PFP. **Appendix F.** Demographic and clinical characteristics of participants with discordant prefrailty/frailty classification by FI and count- or model-based PFP. **Appendix G.** Approximated model equation.

## Data Availability

All data generated or analyzed during this study are included in this published article [and its supplementary information files].
