# Supplementary material for: Development and validation of a physical frailty phenotype index-based model to estimate the frailty index
Source: Diagn Progn Res. 2023 Mar 21;7:5. doi: 10.1186/s41512-023-00143-3 (PMC10029224; doi:10.1186/s41512-023-00143-3)
Supplement: Supplementary file 1 — Additional file 1: Appendix A. 36-Item frailty index. Appendix B. Model implementation details for model-based PFP. Appendix C. Demographic and clinical characteristics of participants with and without one-year follow-up clinical outcomes. Appendix D. Markov Chain Monte Carlo (MCMC) simulation diagnostics. Appendix E. Reclassification of participants by net reclassification index (NRI) with use of gait speed model or model-based PFP versus count-based PFP. Appendix F. Demographic and clinical characteristics of participants with discordant prefrailty/frailty classification by FI and count- or model-based PFP. Appendix G. Approximated model equation. [file 41512_2023_143_MOESM1_ESM.docx]

| **Supplementary Materials and Methods** | |
| --- | --- |
| Appendix A: | 36-item Frailty Index |
| Appendix B: | Model implementation details for model-based PFP |
| Appendix C: | Demographic and clinical characteristics of participants with and without one-year follow-up clinical outcomes |
| Appendix D: | Markov Chain Monte Carlo (MCMC) simulation diagnostics |
| Appendix E: | Reclassification of participants by net reclassification index (NRI) with use of gait speed model or model-based PFP versus count-based PFP |
| Appendix F: | Demographic and clinical characteristics of participants with discordant prefrailty/frailty classification by FI and count- or model-based PFP. |
| Appendix G: | Approximated model equation |
|  |  |

**Appendix A**: 36-item Frailty Index

| **Variables** | **Cut Point** |
| --- | --- |
| **Functional/ Physical**  Help bathing  Help with transfers  Help walking on level surfaces  Help feeding  Help grooming  Help with toileting  Help on stairs  Bowel incontinence  Bladder incontinence  Help shopping  Help with housekeeping  Help with meal preparation  Help with finances  Help taking medication  Physical activity level  Cut down on usual activity (in last month)  Stayed in bed > half the day due to health (in last month)  Falls in last 1 year  Problems due to poor hearing  Problems due to poor vision  **Psychological/ Cognitive**  Memory changes  Feeling sad  History of depression  Self-rating of health  How health has changed in last year  **Social**  Living alone?  Having someone to talk to when lonely  **Medical**  Symptoms  Pain  Weakness  Co-morbidities  Hypertension  Ischemic heart disease  Diabetes mellitus  Chronic lung disease  Arthritis  Body Mass Index (BMI)  Unintentional weight loss >5% past 6 months | Dependent=1, Some assistance=0.5, Independent=0  Dependent=1, Some assistance=0.5, Independent=0  Unable=1, Some assistance=0.5, Independent=0  Dependent=1, Some assistance=0.5, Independent=0  Dependent=1, Some assistance=0.5, Independent=0  Dependent=1, Some assistance=0.5, Independent=0  Unable=1, Some assistance=0.5, Independent=0  Yes=1, No=0  Yes=1, No=0  Unable=1, Some assistance=0.5, Independent=0  Unable=1, Some assistance=0.5, Independent=0  Unable=1, Some assistance=0.5, Independent=0  Unable=1, Some assistance=0.5, Independent=0  Unable=1, Some assistance=0.5, Independent=0  <4 hours activity weekly=1, >4 hours activity weekly=0  Yes=1, No=0  Yes=1, No=0  >2 falls=1, 1 to 2 falls=0.5, No fall=0  Yes=1, No=0  Yes=1, No=o  Yes=1, No=0  Yes=1, No=0  Yes=1, No=0  Poor=1, Fair=0.75, Good=0.5, V good=0.25, Excellent=0  Worse=1, Better/ same=0  Yes=1, No=0  Yes=0, No=1  Yes=1, No=0  Yes=1, No=0  Yes=1, No=0  Yes=1, No=0  Yes=1, No=0  Yes=1, No=0  Yes=1, No=0  <18.5=1, 18.5 to 23 =0, 23-27.5 = 0.5, >27.5 = 1  Yes=1, No=0 |

**Appendix B:** Model implementation details for model-based PFP

| Formula | Mean model  frailty_index_beta_transformed ~ men +  s(handgrip_z, k=4, bs="tp") + s(gait_speed_z, k=4, bs="tp") +  s(body_weight_z, k=4, bs="tp") + s(body_height_z, k=4, bs="tp") +  s(walktime_weekly_cuberoot_transformed, k=4, bs="tp") +  mo(exhaustion_q1) + mo(exhaustion_q2)  Precision model  phi ~ men + body_weight_z + body_height_z |
| --- | --- |
| Family | Beta(link="logit") |
| Priors | Intercept mean model: student_t(nu = 3, mu = 0, sigma = 2.5)  Intercept precision model: student_t(nu = 3, mu = 0, sigma = 2.5)  Betas (slopes) mean model: normal (mu=0, sigma = 0.85)  Betas (slopes) precision model : student_t(nu = 3, mu = 0, sigma = 0.39)  Monotonic effects: dirichlet(alpha = 1)  Smoothing effects SD: student_t(nu = 3, mu = 0, sigma = 1) |
| Hyperparameters | Delta (target acceptance rate) = 0.995  Number of Markov chains = 4  Warmup iterations per chain = 1000  Inference iterations per chain = 3000 |

***Model and prior specification***

To develop the model-based PFP, we fitted a Bayesian multivariable beta regression model, which included (i) FI as the response variable and (ii) PFP component criteria and sex as predictors. A Bayesian analytical framework was used because it aligned closely with our objectives of (i) modelling the 2 PFP “exhaustion” criterion items flexibly as monotonic ordered predictors^1^ and (i) providing interpretable uncertainty estimates of the predicted FI values (both described later). Beta regression was used because it is a flexible approach to model the FI – a continuous proportion with a non-normal distribution^2^. In our study, only 9 participants (~1%) had a FI of 0 and the probability of zero values is not of substantive clinical interest. Hence, to meet the range requirement of beta regression, we used the formula by Smithson and Verkuilen ^2^ to transform the response variable within the 0-to-1 bounds. To account for heteroscedasticity and potential sex and body size differences in the variability of FI, we modeled the dispersion of the beta distribution to vary with sex, body weight, and height.

Our goal was to optimize the predictive accuracy of the PFP by preserving information in its criterion predictors. Thus, gait speed, handgrip strength, body weight, body height, and total walking time were treated as continuous variables. For total walking time, this variable was first transformed using its cube root to reduce the potential influence of extreme values. To allow prior distributions (described later) to be scaled for other predictors, we standardized them as *z*-scores. To avoid assuming linearity for all continuous predictors, we modelled them with thin-plate splines using 4 basis functions (*k*=4)^3^. For the 2 "exhaustion" variables, we modelled these ordinal predictors using the "monotonic effects" function^1^ which estimates (i) the average (expected) difference in FI between 2 adjacent ordinal categories and (ii) the expected normalized difference in FI between each pair of the adjacent ordinal categories. Hence, this analytical approach allows ordinal categories to exert individual conditional effects whilst maintaining monotonically (same directionality).

In our analyses, we set weakly-informative prior distributions for the model parameters to reduce the likelihood of estimating unrealistic values without excluding reasonable values^4^. Prior specifications are as follows: we set normal priors (μ=0, σ=0.89) for the beta regression weights, normal priors (μ=0, σ=0.39) for the dispersion regression weights, non-negative (half) Student's *t* priors (υ=3, μ=0, σ=1) for the standard deviation of smoothing effects, and Dirichlet priors (α=1) for the monotonic effects of ordinal predictors. For all other model parameters, we used default non-informative (flat) priors to reflect our lack of in-depth information about them.

All Bayesian models were fitted using *Stan*^5^ through the *brms*^6^ R package. Stan implements the Hamiltonian Monte Carlo with No-U-Turn sampling algorithm^5^, and each model used 4 chains, 3000 iterations per chain, to generate the posterior samples for all parameters. From these samples, we derived the posterior predictive distribution of the FI which could be interpreted as the predictions of possible mean FI values for a given individual characterized by a given set of PFP criterion values. To summarize this distribution, we used mean as point estimate and 95% credible interval (CrI) as the interval with 95% probability of containing the true FI, given our prior knowledge and observed data.

***References***

1. Bürkner PC, Charpentier E. Modelling monotonic effects of ordinal predictors in Bayesian regression models. *Br J Math Stat Psychol*. 2020;73(3):420-451. doi:10.1111/bmsp.12195

2. Smithson M, Verkuilen J. A better lemon squeezer? Maximum-likelihood regression with beta-distributed dependent variables. *Psychol Methods*. 2006;11(1):54-71. doi:10.1037/1082-989X.11.1.54

3. Wood SN. Thin plate regression splines. *Journal of the Royal Statistical Society: Series B (Statistical Methodology)*. 2003;65(1):95-114. doi:10.1111/1467-9868.00374

4. Gelman A, Jakulin A, Pittau MG, Su YS. A weakly informative default prior distribution for logistic and other regression models. *Ann Appl Stat*. 2008;2(4):1360-1383.

5. Stan Development Team. RStan: the R interface to Stan. *R package version*. 2021;2.21.3. https://mc-stan.org/

6. Bürkner PC. brms: An R package for Bayesian multilevel models using Stan. *Journal of statistical software*. 2017;80(1):1-28.

**Appendix C**: Demographic and clinical characteristics of participants with and without one-year follow-up clinical outcomes

| Appendix Table F: Demographic and clinical characteristics of participants with and without follow-up outcomes. | | |
| --- | --- | --- |
| **Variables** | **Participants with outcomes (n=572)** | **Participants without outcomes (n=512)** |
| Age (years) | 64 **68** 73  (68.4 ± 6.9) | 62 **67** 71  (67.0 ± 6.8) |
| Women | 77% (442) | 70% (358) |
| Weight (kg) | 51.6 **57.7** 66.6  (59.2 ±11.6) | 52.9 **60.9** 69.1  (61.6 ±11.9) |
| Height (m) | 1.51 **1.55** 1.60  (1.56 ±0.08) | 1.51 **1.56** 1.63  (1.57 ±0.08) |
| BMI (kg/m^2^) | 21.7 **23.7** 26.3  (24.3 ± 4.4) | 21.9 **24.3** 27.6  (24.9 ± 4.6) |
| Hypertension | 44% (253) | 46% (235) |
| Diabetes mellitus | 21% (121) | 21% (105) |
| Depressive Symptoms | 15% ( 85) | 17% ( 87) |
| Arthritis | 20% (112) | 15% ( 76) |
| Ischaemic Heart Disease | 5% ( 26) | 2% ( 9) |
| Stair climbing difficulty | 18% (102) | 15% ( 76) |
| Lifting (10pounds) difficulty | 18% (101) | 15% ( 77) |
| Frailty Index (FI) | 0.07 **0.10** 0.17  (0.12 ±0.08) | 0.06 **0.10** 0.15  (0.11 ±0.07) |
| FI classification |  |  |
| Robust (<0.10) | 48% (273) | 53% (270) |
| Prefrail (0.10-0.21) | 41% (234) | 38% (193) |
| Frail (>0.21) | 11% ( 65) | 10% ( 49) |
| Count-based PFP |  |  |
| Robust (0pts) | 63% (358) | 67% (343) |
| Prefrail (1-2pts) | 34% (192) | 30% (153) |
| Frail (3-5pts) | 4% ( 22) | 3% ( 16) |
| Model-based PFP |  |  |
| Predicted FI | 0.08 **0.10** 0.13  (0.12 ±0.05) | 0.08 **0.10** 0.13  (0.11 ±0.04) |
| Robust (<0.10) | 47% (266) | 47% (243) |
| Prefrail (0.10-0.21) | 48% (273) | 48% (244) |
| Frail (>0.21) | 6% ( 33) | 5% ( 25) |
| Continuous variables are summarized as 25^th^, **50**^th^, 75^th^ percentiles (mean ± SD). Categorical variables are summarized as percentages and frequencies (*N*).  FI = frailty index; PFP = physical frailty phenotype  * Geriatric Depression Scale (GDS) ≥5 points | | |

**Appendix D**: Markov Chain Monte Carlo (MCMC) simulation diagnostics

The Figures below show the posterior distributions for all model parameters (left column) and the trace plots of the Markov-Chain Monte-Carlo simulations (right column). The trace plots show stable convergence.


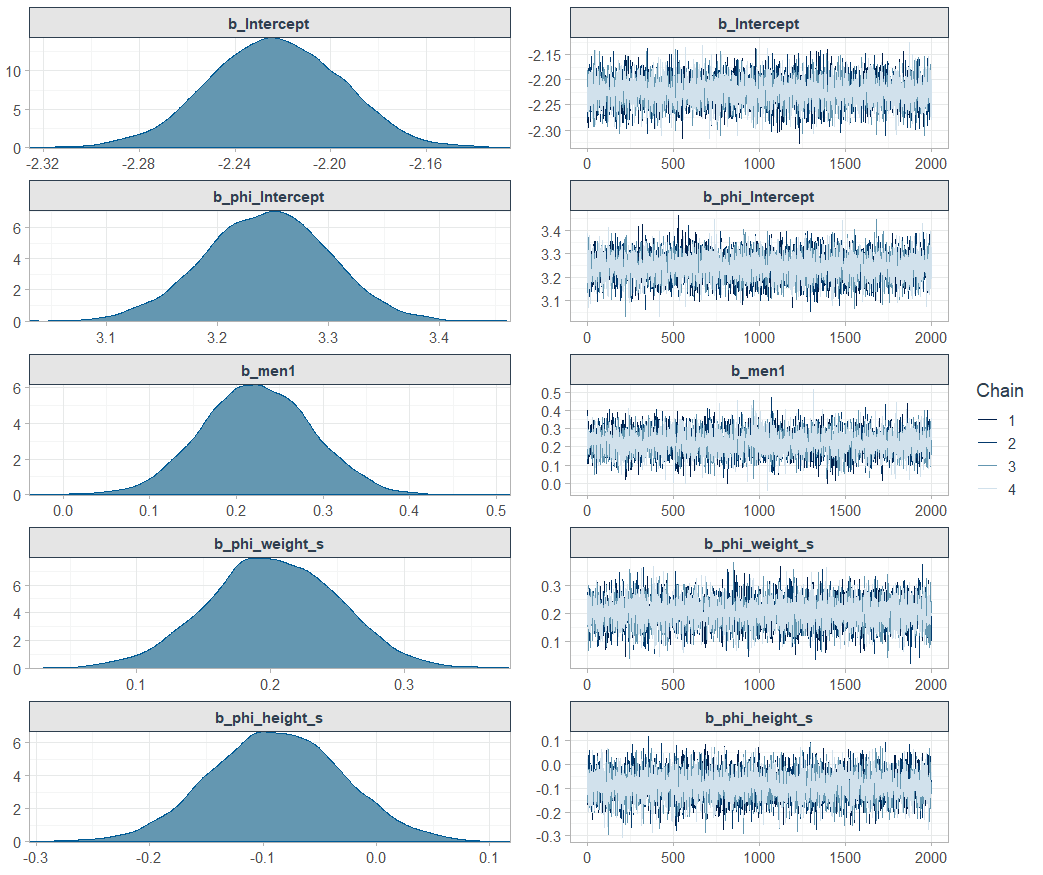


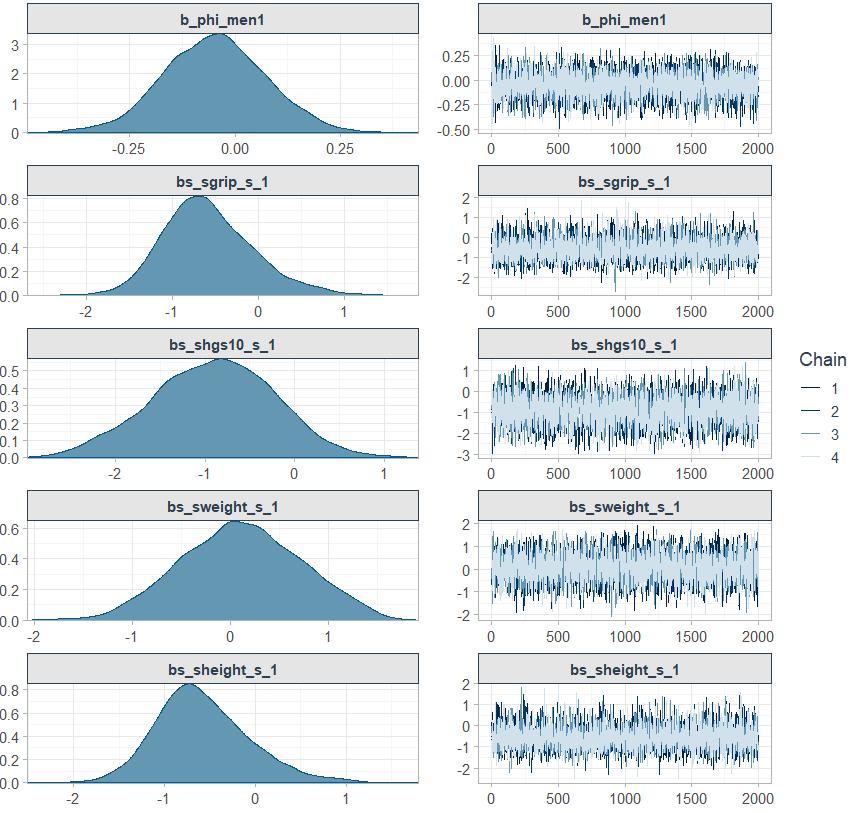

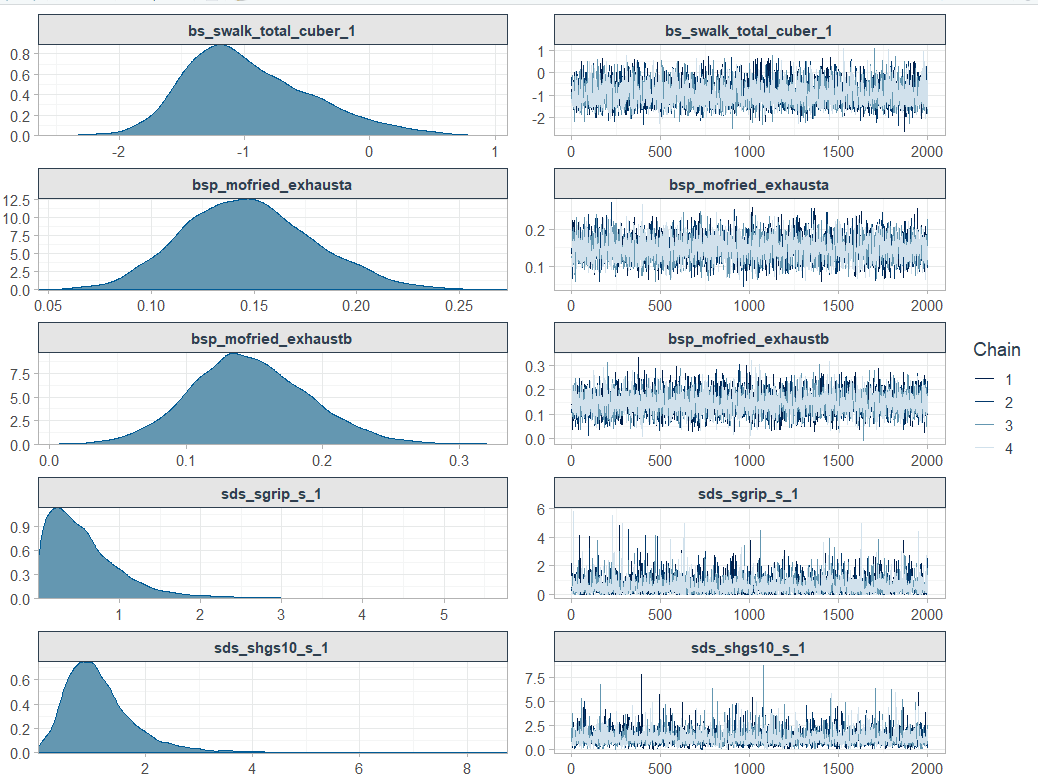


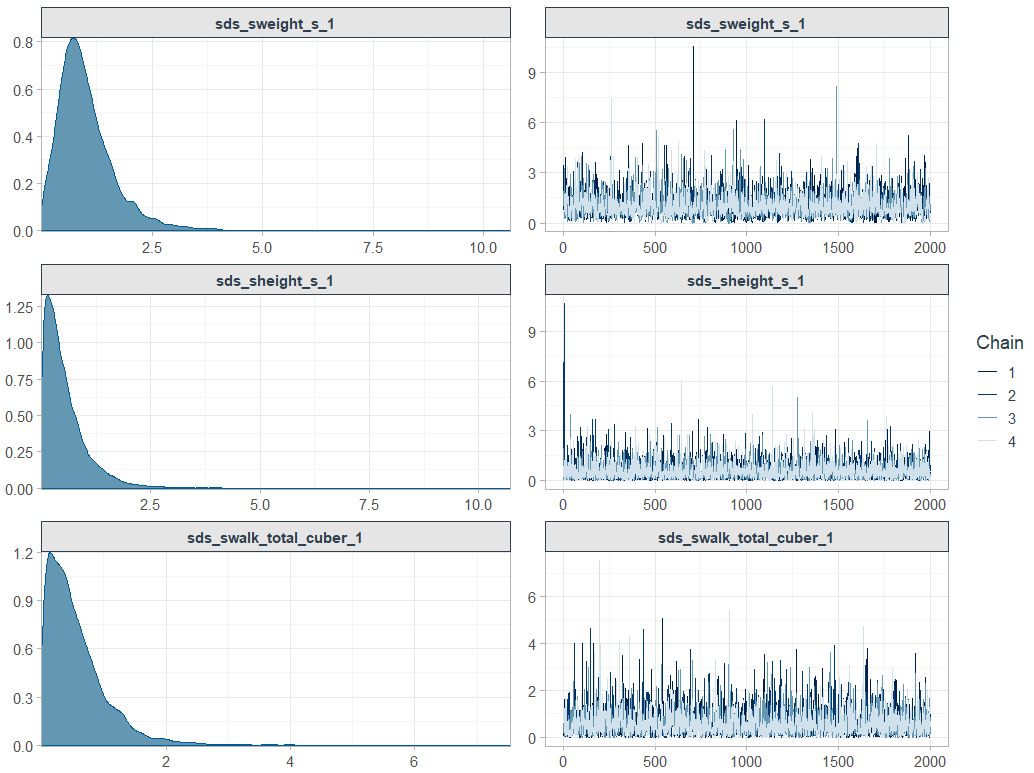


**Appendix E**: Reclassification of participants by net reclassification index (NRI) with use of gait speed model or model-based PFP versus count-based PFP

| Appendix D: Reclassification of Participants by NRI with use of gait speed model or model-based PFP versus count-based PFP^†^ | | | | | | | | | |
| --- | --- | --- | --- | --- | --- | --- | --- | --- | --- |
|  | Prefrail/Frail Participants | | | |  | Robust Participants | | | |
| Model | Participants n | Correctly Reclassified n(%) | Incorrectly Reclassified n(%) | NRI event |  | Participants n | Correctly Reclassified n(%) | Incorrectly Reclassified n(%) | NRI  non-event |
| Gait speed | 484 | 143 (0.30) | 48 (0.10) | 0.20 |  | 514 | 56 (0.11) | 130 (0.25) | -0.14 |
| Model-based PFP | 484 | 133 (0.27) | 23 (0.05) | 0.23 |  | 514 | 45 (0.09) | 104 (0.20) | -0.11 |
| † Prefrailty/Frailty is defined by Frailty Index > 0.10 | | | | | | | | | |

**Appendix F**: Demographic and clinical characteristics of participants with discordant prefrailty/frailty classification by FI and count- or model-based PFP.

| **Appendix F1.** Demographic and clinical characteristics of participants with discordant prefrailty/frailty classification by FI and count-based PFP. | | | | |
| --- | --- | --- | --- | --- |
| **Variables** | **Prefrail/Frail by count PFP but not by FI (*n*=122)** | | **Prefrail/Frail by FI but not by count PFP (*n* =228)** | ***P*-value** |
| Age (years) | | 63.0 68.0 72.0  (67.9 ± 6.7) | 63.0 67.0 71.0  (67.6 ± 6.4) | 0.57^1^ |
| BMI (kg/m^2^) | | 19.9 22.5 25.1  (22.9 ± 4.3) | 23.1 25.1 28.4  (26.0 ± 4.1) | <0.001^1^ |
| Men | | 25% ( 31) | 26% ( 59) | 0.92^2^ |
| Depressive Symptoms | | 2% ( 2) | 22% ( 51) | <0.001^2^ |
| Arthritis | | 7% ( 9) | 26% ( 59) | <0.001^2^ |
| Ischaemic Heart Disease | | 1% ( 1) | 4% ( 9) | 0.09^2^ |
| Stair climbing difficulty | | 7% ( 8) | 15% ( 34) | 0.02^2^ |
| Lifting (10pounds) difficulty | | 8% ( 10) | 18% ( 42) | 0.01^2^ |
| Continuous variables are summarized as 25^th^, **50**^th^, 75^th^ percentiles (mean ± SD), and tested with the Wilcoxon Mann Whitney test^1^. Categorical variables are summarized as percentages and frequencies (*N*), and tested with the Pearson's χ^2^ test^2^. | | | | |

| **Appendix F2**. Demographic and clinical characteristics of participants with discordant prefrailty/frailty classification by FI and model-based PFP. | | | | |
| --- | --- | --- | --- | --- |
| **Variables** | **Prefrail/Frail by Model-based PFP but not by FI (*n*=181)** | | **Prefrail/Frail by FI but not by model-based PFP (*n*=118)** | ***P*-value** |
| Age (years) | | 62.0 68.0 73.0  (67.7 ± 6.7) | 62.0 66.0 71.0  (66.8 ± 6.4) | 0.21^1^ |
| BMI (kg/m^2^) | | 22.5 25.1 27.9  (25.4 ± 4.5) | 20.7 23.3 25.3  (23.3 ± 3.6) | <0.001^1^ |
| Men | | 28% ( 51) | 19% ( 23) | 0.09^2^ |
| Depressive Symptoms | | 2% ( 4) | 24% ( 28) | <0.001^2^ |
| Arthritis | | 9% ( 16) | 28% ( 33) | <0.001^2^ |
| Ischaemic Heart Disease | | 0% ( 0) | 4% ( 5) | 0.005^2^ |
| Stair climbing difficulty | | 9% ( 16) | 8% ( 9) | 0.71^2^ |
| Lifting (10pounds) difficulty | | 9% ( 17) | 15% ( 18) | 0.12^2^ |
| Continuous variables are summarized as 25^th^, **50**^th^, 75^th^ percentiles (mean ± SD), and tested with the Wilcoxon Mann Whitney test^1^. Categorical variables are summarized as percentages and frequencies (*N*), and tested with the Pearson's χ^2^ test^2^. | | | | |

**Appendix G**: Approximated model equation

To promote greater transparency in our algorithm, we have constructed an approximated model equation by regressing the posterior predicted FI estimates on the predictors in a (Frequentist) linear regression model. In that model, continuous predictors are expanded as restricted cubic splines (with 3 knots) whilst ordinal predictors are modelled using quadratic terms. The resultant model has an adjusted *R*-squared value of 0.98, and its equation, generated using Harrell's *Hmisc* *R* package [1], takes the form:


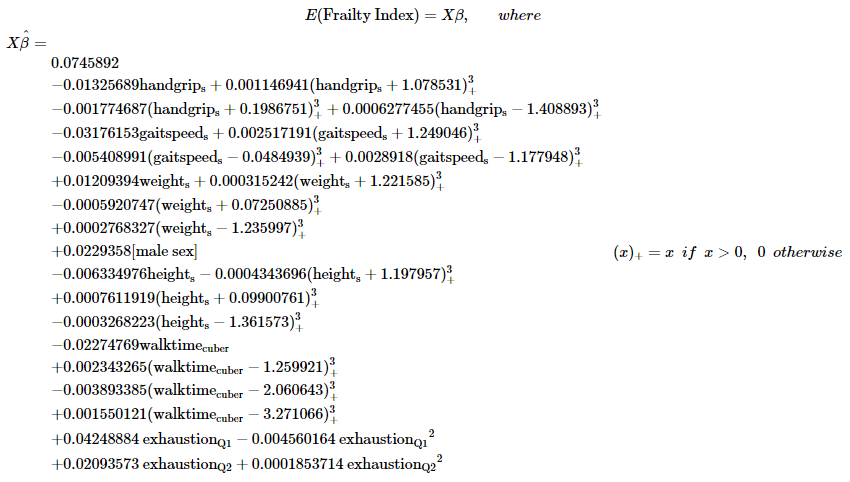


Reference

[1] Harrell Jr FE. Hmisc: Harrell Miscellaneous. *R* package version 4.7-0. https://cran.project.org/web/packages/Hmisc/index.html.
